# An Incus-Body Driving Type Piezoelectric Middle Ear Implant Design and Evaluation in 3D Computational Model and Temporal Bone

**DOI:** 10.1155/2014/121624

**Published:** 2014-06-18

**Authors:** Houguang Liu, Zhushi Rao, Xinsheng Huang, Gang Cheng, Jiabin Tian, Na Ta

**Affiliations:** ^1^School of Mechatronic Engineering, China University of Mining and Technology, Xuzhou 221116, China; ^2^State Key Laboratory of Mechanical System and Vibrations, Shanghai Jiaotong University, Shanghai 200240, China; ^3^Department of Otorhinolaryngology, Zhongshan Hospital, Fudan University, Shanghai 200032, China

## Abstract

A new incus-body driving type transducer relying on piezoelectric stack, with broad frequency bandwidth, is proposed for use in a middle ear implant. To aid the design process of this transducer, a coupling biomechanical model of the human middle ear and the piezoelectric transducer was established by reverse engineering technology. The validity of this model was confirmed by comparing model predicted motions with experimental measurements. Based on this verified biomechanical model, the main parameters of the transducer were determined. And its power consumption was calculated. Finally, to verify the capability of the designed piezoelectric transducer, a human temporal bone experimental platform was built. And the dynamic characteristics and the stimulated performance of the piezoelectric transducer were tested. The result showed that stapes displacement stimulated by the transducer excitation at 10.5 V RMS was equivalent to that from acoustic stimulation at 100 dB SPL, which is an adequate stimulation to the ossicular chain. The corresponding power consumption is 0.31 mW per volt of excitation at 1 kHz, which is low enough for the transducer to be used in a middle ear implant. Besides, this transducer demonstrates high performance at high frequencies.

## 1. Introduction

There are estimated 27 million people in China with hearing loss. Worldwide, the number is estimated to be more than 500 million. With the development of ear surgery, most of the conductive hearing loss can benefit from operation, whereas there is still lack of effective treatment for sensorineural hearing loss. The majority of these hearing-impaired individuals can only turn to traditional hearing aids. However, traditional hearing aids have several inherent disadvantages, such as higher sound distortion, limited amplification, noise and ringing, discomfort, and cosmetic appearance [1].

To overcome these shortcomings of traditional hearing aids, middle ear implant (MEI) becomes a dynamic area of research [2–4]. Unlike conventional hearing aids which use amplified sound pressure to compensate hearing impairment, MEI always takes advantage of direct mechanical stimulation to compensate hearing loss. This mechanical stimulation is accomplished by an implanted transducer. From 1990, several types of MEIs equipped with electromagnetic or piezoelectric transducer have been investigated or developed in the world [5, 6]. Among them, the incus-body driving type MEI is one of the widely investigated types for its minor damage to the structure of the middle ear system [7–9]. Up to now, this type of MEI uses either an electromagnetic transducer or a piezoelectric diaphragm transducer as its vibrator. Comparatively, the piezoelectric diaphragm transducer has demonstrated many advantages including ease of fabrication, wider bandwidth, lower power consumption, and more compatibility with external magnetic environment. However, it cannot generate enough stimulating displacement at high frequencies to compensate severe sensorineural hearing loss owing to its specific structure.

Accordingly, in this paper, a piezoelectric stack (piezostack) transducer for incus-body driving type MEI was proposed. This piezostack transducer conserved all the advantages of the piezoelectric diaphragm transducer. Besides, it has a high output gain at high frequencies. To aid the design process of the transducer, a coupling biomechanical model of the transducer and the middle ear was established and verified. And the piezostack's dimension and the layer number were ascertained so that this transducer can generate a large excitation force, which is sufficient for application in patients with severe sensorineural hearing loss. Finally, a prototype of this transducer was made and its fundamental properties were examined by a temporal bone experiment.

## 2. Materials and Methods

### 2.1. Design of the Device

**Figure 1:**
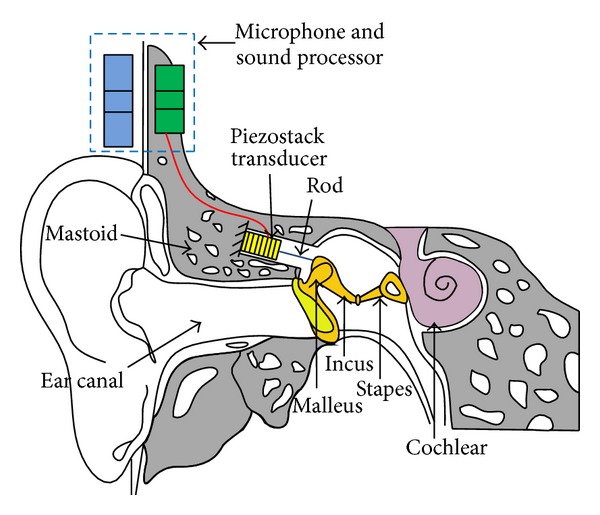
Schematic of the proposed middle ear implant.

The schematic of the proposed MEI is illustrated in Figure 1. This MEI includes a microphone for receiving an acoustic signal from outside, a sound processor for receiving the acoustic signal from the microphone to generate an acoustic electric signal, and a piezostack transducer for generating a vibration in response to the acoustic electrical signal from the sound processor. One side of the piezostack is fixed to the mastoid, which is a bony structure behind the external auditory canal, while the other side is stuck to the incus body using a coupling rod. Thus, the incus can be vibrated by the repetition of the piezostack transducer's expansion and contraction according to the applied voltage. Then, the vibration is transferred to the cochlea, where the mechanical movement is transformed into a neural response.

#### 2.1.1. Dimensions of the Piezostack

The incus-body driving type piezostack transducer is surgically implanted in the mastoid. Due to the confined space of the mastoid, the dimensions of the transducer are limited. However, small dimensions will increase the manufacturing process requirement. Considering these two factors, the piezostack's cross section is set to 2 × 2 mm^2^ with thickness of 2 mm. And the piezostack is made up of the lead zirconate titanate (PZT-4) ceramics considering its common use and low cost.

#### 2.1.2. Layer Number of the Piezostack

As the human middle ear is a tiny but complex structure, it is difficult to investigate the effect of the piezostack's layer number's change on the transfer function of the middle ear using temporal bones experiment. Finite element (FE) method is capable of easily modeling the complex geometry, ultrastructural characteristics, and nonhomogenous and anisotropic material properties of human ear [10, 11]. Thus, we first constructed a middle ear finite element model. Then, we took advantage of this FE model to ascertain the required piezostack's layer number.

### 2.2. Middle Ear Finite Element Model

**Figure 2:**
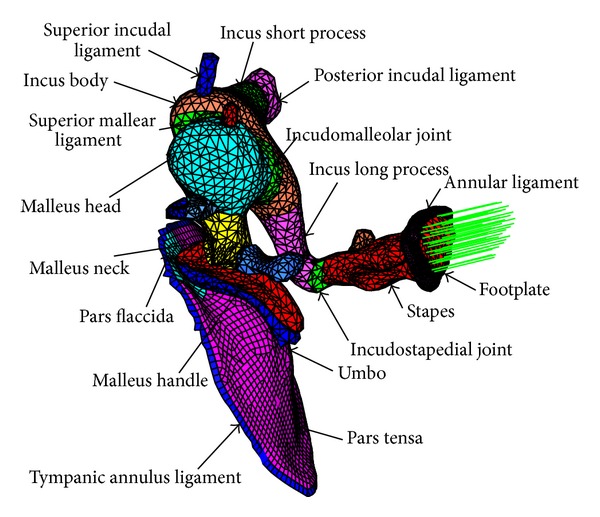
Human middle ear finite element model.

To facilitate the design of the piezostack's layer number, a middle ear FE model was constructed as shown in Figure 2. This finite element model was established based on a complete set of microcomputerized tomography section images of a fresh human temporal bone by reverse engineering technology [12]. The material properties and boundaries of this model were listed in Tables 1 and 2. These material property values in Table 1 were taken from literature reports [13–17], and the boundary conditions in Table 2 were taken from papers [13, 15, 16]. Besides, the Poisson ratio was assumed to be 0.3 for all materials of the middle ear system, and the damping parameters were assumed to be *α* = 0 s^−1^ and *β* = 0.0001 s [17].

In order to establish the extent to which the prediction of the middle ear finite element model accords with reality, comparisons against two experimental studies were done. Aibara et al. [18] experimental data of stapes footplate velocity transfer function (STF) obtained from 11 fresh temporal bones were initially selected for the model verification. Their measurements were conducted using a laser Doppler interferometer. When a pure tone narrow band filtered sound of 90 dB SPL was delivered near the eardrum in the ear canal, the velocity of the stapes footplate was measured, and the STF was obtained. For comparison, a uniform pressure of 90 dB SPL on the lateral side of the eardrum was applied to our middle ear FE model. A harmonic analysis was conducted on the model across the frequency range of 160–8,000 Hz. Then, the STF was calculated according to the following equation:

(1) $$\begin{array}{c} S_{\text{TF}}=\frac{\upsilon }{P_{\text{TM}}},\;\upsilon =2\pi fD, \end{array}$$

where *υ* is footplate velocity, *P*_TM_ is sound pressure at umbo, *f* is frequency, and *D* is footplate displacement.

The experimental data of umbo displacement published by Nishihara et al. [19] were also selected for our model evaluation. With a uniform harmonic pressure stimulus of 80 dB SPL applied to the lateral side of the eardrum in our middle ear FE model, a harmonic analysis was conducted across the frequency range of 160–8,000 Hz.

### 2.3. Coupling Mechanical Model of the Middle Ear and the Piezostack

To investigate the effect of the piezostack's layer number's change on the transfer function of the middle ear, a coupling biomechanical model of the middle ear and the piezostack was built. Firstly, the piezoelectric transducer's piezostack finite element model was constructed. The material properties of the PZT-4 ceramics are listed in Table 3. Its piezoelectric FEM equations can be written in terms of nodal displacement **U** and nodal electrical potential Φ for each node. The mechanical efforts are expressed by **F** and the nodal electric loads by **Q**, resulting in the equilibrium equations below [20]:

(2) $$\begin{array}{c} M_{uu}\ddot{U}+C_{uu}\dot{U}+K_{uu}U+K_{u\varphi }\Phi =F,\\ K_{u\varphi }^{\text{T}}U+K_{\varphi \varphi }\Phi =Q. \end{array}$$

This same expression can also be expressed in the matrix form:

(3) (Muu000)(U¨Φ¨) +(Cuu000)(U˙Φ˙)+(KuuKuφKuφTKφφ)(UΦ)=(FQ),

where

(4) Kuu= ∭ΩeBuTcBudV,Kuφ=∭ΩeBuTeBφdV,Kφφ=∭ΩeBφTεBφdV,Muu=ρ∭ΩeNuTNudV,  Cuu=βKuu,

where  **K**_**uu**_ is mechanical stiffness matrix, **K**_**u***φ*_ is piezoelectric coupling matrix, **K**_*φφ*_ is dielectric stiffness matrix, **M**_**uu**_ is mass matrix, and **C**_**uu**_ is mechanical damping matrix.

**Figure 3:**
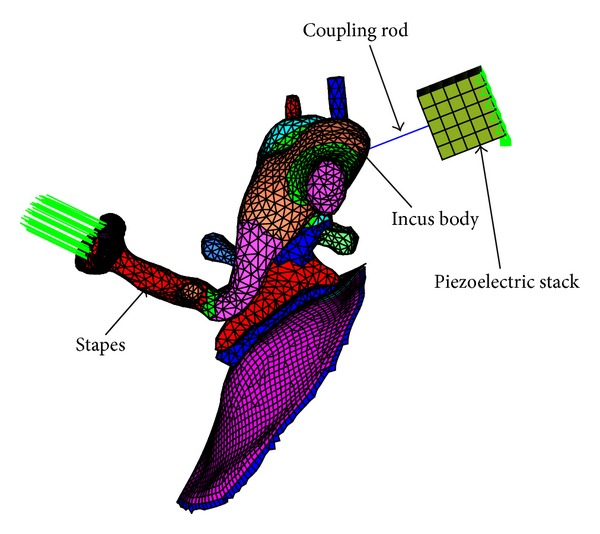
Coupling mechanical model of the piezoelectric stack transducer and the middle ear.

Then this piezostack FE model was coupled with the middle ear FE model as shown in Figure 3. In this model, the coupling rod with 0.5 mm in diameter and 2.0 mm in length was modeled using 6 beam elements. Confining the upper voltage limit of the piezoelectric transducer's FE model to 10.5 V root mean square (RMS) for safety requirements [21], the layer number's influence on the MEI's performance was calculated, and the required layer number of the piezostack was ascertained.

The coupling rod is responsible for transferring the vibration of the piezostack to the middle ear system. To analyze the influence of the coupling rod's stiffness on the transducer's hearing compensation performance, Young's modulus of the coupling rod was varied to 116 GPa (titanium coupling rod) and 510 GPa (ceramic coupling rod). Finally, the effect of the coupling rod on the transducer's performance is signified by the change of the stapes displacement expressed in decibels in the following equation:

(5) $$\begin{array}{c} \Delta H=\text{lo}{\text{g}}_{10}\left(\frac{d_{\mathrm{tr}\_\text{rod}}}{d_{\mathrm{tr}}}\right), \end{array}$$

where **d**_tr⁡_ is stapes displacement excited directly by the piezostack (ignoring the structure of the coupling rod) and **d**_tr⁡_rod_ is transducer excited stapes displacement considering the structure of the coupling rod.

### 2.4. Transducer Fabrication and Measurement

The transducer was fabricated based on the results derived by the FE model. As many of the patients suffer hearing loss at high frequencies (2–8 kHz), the transducer for MEI should have a significant output at this range. To evaluate the practical performance of the fabricated transducer, the vibration displacement of the transducer has been measured by a laser vibrometer (PSV 300, Polytec, Germany).

**Figure 4:**
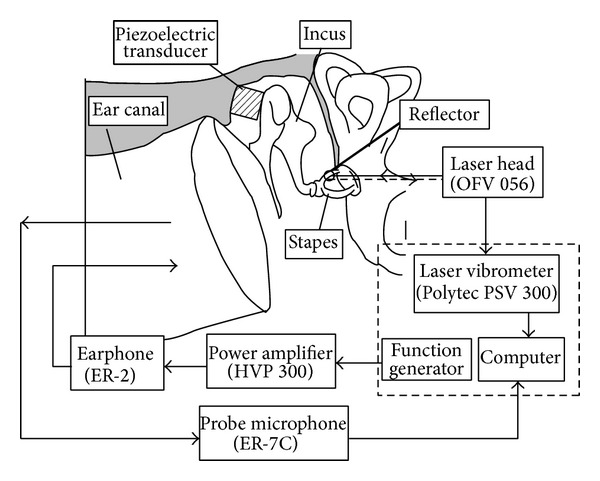
Experiment setup of the measurement system.

To verify the hearing compensation performance of the designed piezostack transducer, a human temporal bone experiment was carried out. For comparison, the normal sound transfer property of the human middle ear was tested firstly. The stapes displacement in response to an acoustic stimulation in front of the eardrum was measured prior to the attachment of the piezoelectric transducer. The experimental setup was shown in Figure 4. A 100 dB SPL acoustic stimulation was applied at the eardrum by an ER-2 earphone (Etymotic Research, USA), and the displacement of the stapes was measured by a laser vibrometer (PSV 300, Polytec, Germany). During the experiment, a probe microphone ER-7C (Etymotic Research, USA) was used to control the sound pressure applied to the eardrum.

**Figure 5:**
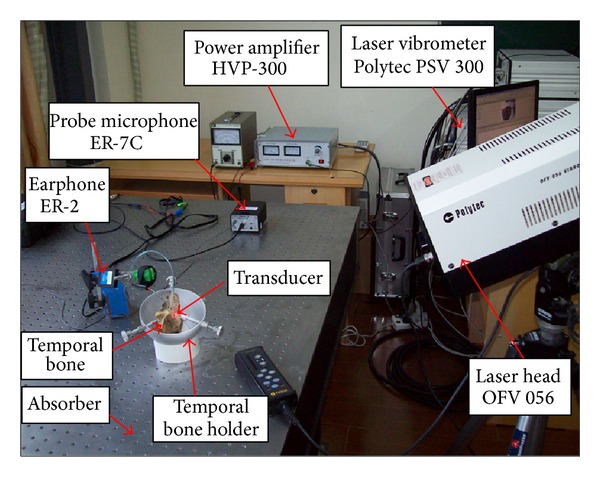
Picture of the temporal bone experiment.

After measuring the stapes displacement under acoustic stimulation, the piezoelectric transducer was attached to the incus body as shown in Figure 4 with epoxy resin. Then, the stapes displacements were measured again by the laser vibrometer when a sine wave driving voltage was applied to the piezostack transducer. The picture of this temporal bone experiment is shown in Figure 5.

### 2.5. Equivalent Sound Pressure Level

In order to facilitate the evaluation of the transducer's performance, the measured stapes displacement stimulated by the piezostack transducer was expressed in terms of the equivalent sound pressure level applied at the tympanic membrane. This equivalent sound pressure level was calculated according to the following equation:

(6) $$\begin{array}{c} P_{\text{eq}}=100+20\times \text{lo}{\text{g}}_{10}\left(\frac{d_{\mathrm{tr}}}{d_{\text{ac}}}\right), \end{array}$$

where **d**_tr⁡_ is stapes displacement under transducer stimulus and **d**_ac_ is stapes displacement under acoustical stimulus of 100 dB SPL.

### 2.6. Power Consumption

The total power consumption is very important for any hearing implantable device. For MEI's transducer, power consumption of 10 mW for an output corresponding to 100 dB SPL at 1 kHz was reported [22]. To ensure our designed transducer is reasonable for MEIs, the power consumption of this transducer was calculated. The behavior of this piezostack transducer can be approximated to that of a capacitor when operated greatly below its resonant frequency [23]. The capacitance *C* can be estimated by summing the capacitances of all the *n* layers as follows:

(7) $$\begin{array}{c} C=n\frac{\varepsilon_{0}\varepsilon_{33}A}{t}, \end{array}$$

where *ε*_0_ is the permittivity in free space, *ε*_33_ is the relative permittivity of the dielectric, *A* is the electrode surface area, and *t* is the single layer thickness.

When excited with a sinusoidal voltage *V*_rms_, the current *I*_rms_ and the power *P*_rms_ at a frequency *f* are given by

(8) $$\begin{array}{c} I_{\text{rms}}=2\pi fCV_{\text{rms}},\;\;P_{\text{rms}}=\frac{1}{\sqrt{2}}I_{\text{rms}}V_{\text{rms}}=\sqrt{2}\pi fCV_{\text{rms}}^{2}.\; \end{array}$$

## 3. Results and Discussion

### 3.1. Middle Ear FE Model Verification

**Figure 6:**
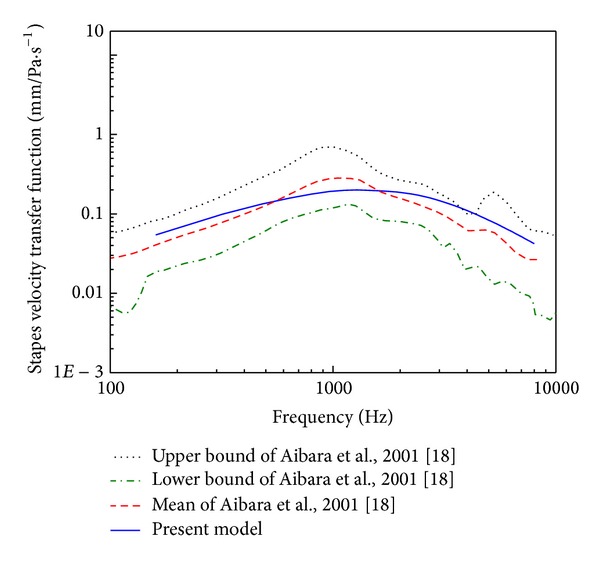
Comparison of the FE model predicted stapes footplate velocity transfer function with the experimental data.

**Figure 7:**
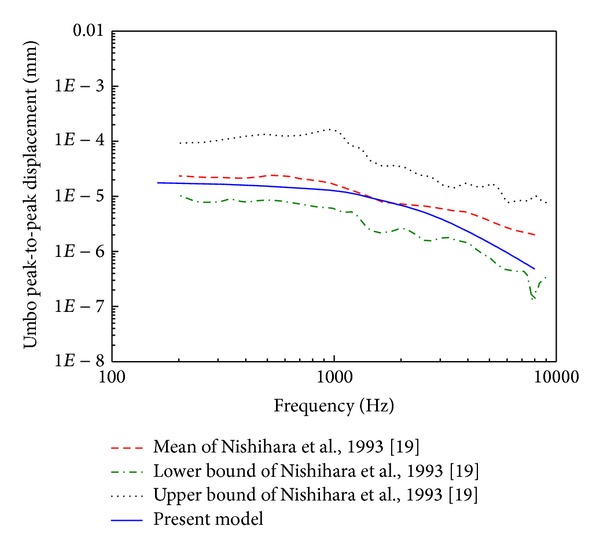
Comparison of the FE model predicted umbo displacement with the experimental data.

The calculated STF was plotted with the mean and the upper and the lower bounds of the eleven experimental curves in Figure 6. It shows that our model predicted STF curve lies close to the mean of the experimental curves. The predicted umbo displacement was plotted with the experimental curves in Figure 7. Likewise, the FE model predicted umbo displacement is close to the mean experimental curve. These above comparisons show that our middle ear FE model's predictions, in general, match experimental results obtained from human temporal bones. Therefore, this middle ear FE model is able to predict biomechanical characteristics of the human middle ear system.

### 3.2. The Influence of the Piezostack's Layer Number

**Figure 8:**
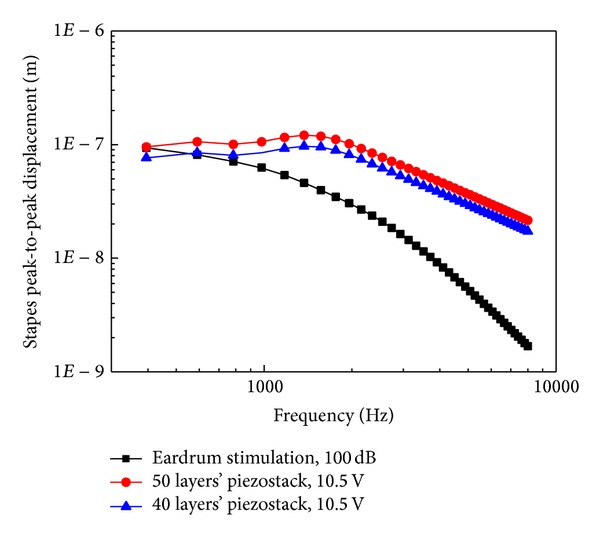
Stapes displacements from acoustic stimulation (100 dB SPL) and piezoelectric stack transducer's stimulation at 10.5 V RMS.

The piezostack's layer number's influence on the transducer's stimulation performance is shown in Figure 8. It shows that stapes displacement from the 50 layers' piezostack transducer excitation at 10.5 V RMS was similar to that from acoustical stimulation at 100 dB SPL at low frequency but considerably greater above 1 kHz. This means that greater level at higher frequencies can be achieved by the 50 layers' transducer excitation at 10.5 V RMS. This level of stapes displacement meets the requirement of a hearing implant [24]. Therefore, the layer number of the transducer's piezostack is set to 50.

### 3.3. The Influence of the Coupling Rod

**Figure 9:**
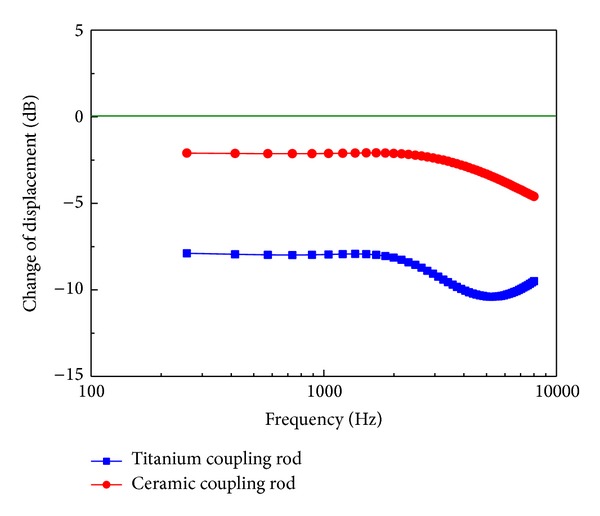
Change of transducer excited stapes displacement with the change of the coupling rod's material.

The effect of the rod's stiffness on stimulated stapes displacement is shown in Figure 9. The result indicates that the insertion of the coupling rod reduces the piezoelectric transducer excited stapes displacement over the entire frequencies. And this decrease appears more significant at higher frequencies (above 2,000 Hz). In addition, this side effect on transducer's performance becomes more serious when the rod's stiffness decreases. The maximum drop in transducer excited stapes displacement is around 10.4 dB at 5 kHz for transducer with titanium coupling rod. The transducer with ceramic coupling rod resulted in a maximum drop of 4.6 dB at 8 kHz.

In addition to the stiffness of the coupling rod, the coupling quality of the rod's tip with the incus body also influences the transducer's performance. Devèze et al. found that the improvement in coupling the transducer to the incus produces significant improvements in the transfer of vibratory stimuli to the ossicular chain [25].

### 3.4. Function of the Piezostack Transducer (Prototype)

**Figure 10:**
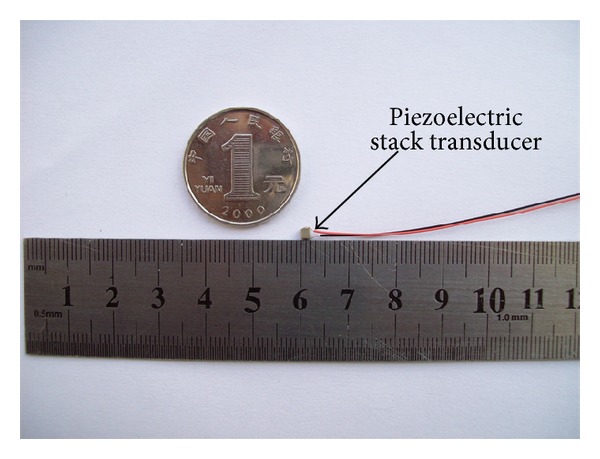
Photograph of the piezostack transducer.

**Figure 11:**
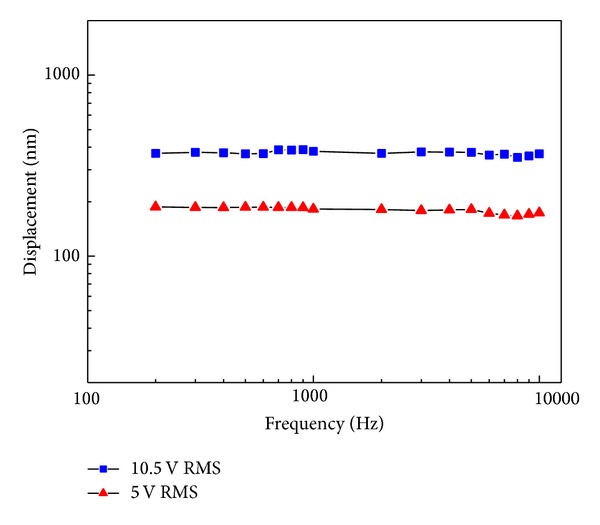
Vibration displacement of the piezoelectric transducer.

As the coupling rod will reduce the piezostack's performance, for the simplification of the experiment, our experimental transducer (prototype) did not contain the coupling rod.  Figure 10 shows the piezostack transducer (prototype) fabricated according to previous sections' design. The transducer is made up of the PZT-4 ceramics and has dimensions of 2 mm × 2 mm × 2 mm. The layer thickness of the piezostack is 0.04 mm and the total number of layers is 50.  Figure 11 shows the measured vibration displacement of this piezostack, when a sine wave driving voltage with a frequency range from 200 Hz to 10 kHz is applied to the transducer. It shows that the frequency characteristic of our transducer is flat up to 10 kHz. Thus, this transducer's performance meets MEI's higher-frequency output's requirement. In addition, Figure 11 also indicates that the transducer's vibration displacement is proportional to its driving voltage. This property will benefit the design of the MEI's signal processor.

**Figure 12:**
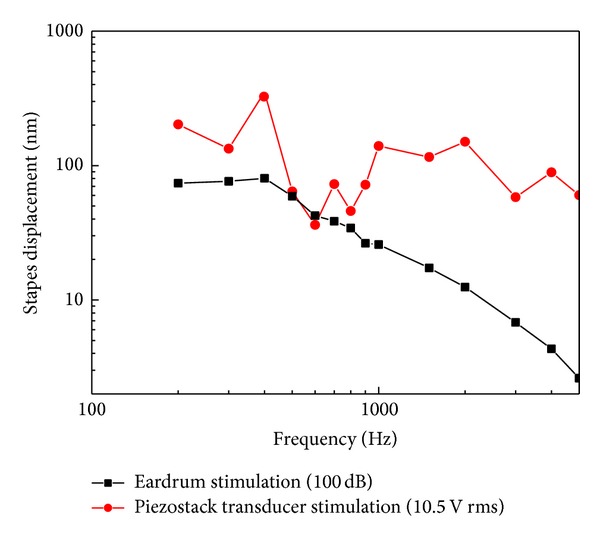
Stapes displacements driven by the piezostack transducer and the acoustic stimulations at the eardrum.

Figure 12 shows the measured stapes displacements driven by the piezoelectric transducer and the acoustic stimulations, respectively. The stapes displacement from transducer excitation at 10.5 V RMS is similar to that from acoustical stimulation at 100 dB SPL at low frequencies (below 1 kHz). Above 1 kHz, the stapes displacement from the transducer excitation was considerably greater than that from acoustic stimulation. The pattern and the trend of these measured stapes displacements' curves are similar to our model's predictions (Figure 8).

### 3.5. Equivalent Sound Pressure Level

**Figure 13:**
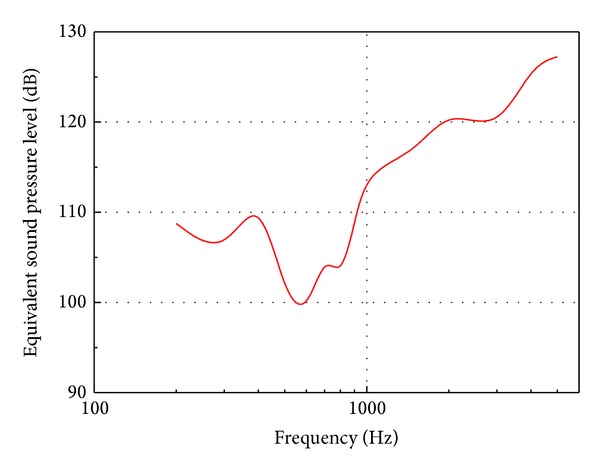
Equivalent sound pressure levels at the tympanic membrane for the transducer excitation at 10.5 V rms.

The transducer stimulated equivalent sound pressure level was plotted in Figure 13. As shown in Figure 13, the transducer driven by 10.5 V RMS can generate about 100 dB SPL equivalent sound pressures at the tympanic membrane below 1 kHz, 120 dB at 2 kHz, and 128 dB above 5 kHz. Given that many MEIs' studies set 100 dB SPL as a design criterion [26], this transducer's performance is sufficient for a MEI's transducer. Moreover, the result also shows that the transducer performs better at high frequency which is a valuable aspect of its performance, considering that the most commonly encountered pattern of sensorineural hearing loss affects the high frequencies more than the low frequencies.

### 3.6. Power Consumption of the Transducer

Our designed PZT-4 piezostack transducer has a capacitance of 71 nF, which resulted in a root mean square current consumption of 0.44 mA and a power consumption of about 0.31 mW per volt of excitation at 1 kHz. Using this transducer at 10.5 V rms to produce a 100 dB SPL equivalent sound pressure at 1 kHz, the power consumption is about 3.26 mW.

According to Zenner's report [27], Ball's invented electromagnetic transducers have a power consumption of about 1.5 W to produce 100 dB SPL equivalent sound pressure at 1 kHz [28]. To stimulate the same level of equivalent sound pressure level at 1 kHz, Maniglia et al. [22] proposed that contactless electromagnetic transducer showed the power consumptions of about 10 mW. Thus, the corresponding power consumption of our piezoelectric transducer, which is 3.26 mW, is much less than that of these reported electromagnetic transducers and reasonable for implantable hearing aids.

## 4. Conclusion

In this paper, a new piezoelectric transducer for the middle ear implant was proposed and designed with the desired frequency characteristics. In consideration of the confined space of the mastoid, this piezoelectric transducer's cross section was set to 2 × 2 mm^2^ with thickness of 2 mm. The layer number of the piezostack was set to 50 by the calculation of a coupling mechanical model of the human middle ear and the piezoelectric transducer.

The designed piezoelectric transducer has a power consumption of 0.31 mW per volt of excitation at 1 kHz, which is reasonable for implantable hearing aids. The temporal bone experiment confirmed that the transducer can generate a vibration force equivalent to a sound pressure of about 100 dB SPL up to 5 kHz. Moreover, it performs better at high frequencies which is a valuable aspect of its performance, given that the most commonly encountered pattern of sensorineural hearing loss affects the high frequencies more than the low frequencies.

## Figures and Tables

**Table 1:** Material properties of the ear components.

Structure	Young's modulus (N/m^2^)	Density (kg m^−3^)
Eardrum		
Pars flaccida	3.20 × 10^7^	1.20 × 10^3^
Pars tensa	1.00 × 10^7^	1.20 × 10^3^
Ossicles		
Malleus		
Head	1.41 × 10^10^	2.55 × 10^3^
Neck	1.41 × 10^10^	4.53 × 10^3^
Handle	1.41 × 10^10^	3.70 × 10^3^
Incus		
Body	1.41 × 10^10^	2.36 × 10^3^
Short process	1.41 × 10^10^	2.26 × 10^3^
Long process	1.41 × 10^10^	5.08 × 10^3^
Stapes	1.41 × 10^10^	2.20 × 10^3^
Joint		
Incudomalleolar joint	1.41 × 10^10^	2.39 × 10^3^
Incudostapedial joint	4.00 × 10^6^	1.20 × 10^3^

**Table 2:** Boundary conditions of the middle ear FE model.

Soft tissue structure	Young's modulus (N/m^2^)	Density (kg m^−3^)
Tympanic annulus ligament	4.00 × 10^5^	1.20 × 10^3^
Superior mallear ligament	4.90 × 10^6^	1.20 × 10^3^
Lateral mallear ligament	6.70 × 10^6^	1.20 × 10^3^
Anterior mallear ligament	2.10 × 10^7^	1.20 × 10^3^
Superior incudal ligament	4.90 × 10^6^	1.20 × 10^3^
Posterior incudal ligament	6.50 × 10^6^	1.20 × 10^3^
Stapedial annulus ligament	4.10 × 10^5^	1.20 × 10^3^
Tensor tympani tendon	2.60 × 10^6^	1.20 × 10^3^
Stapedial tendon	5.20 × 10^5^	1.20 × 10^3^

**Table 3:** Material parameters of the PZT-4 ceramics.

Elastic stiffness constant (GN*·*m^−2^)						Piezoelectric constant (C/m^2^)			Permittivity constant (F/m)	
*c*_11_^*E*^	*c*_12_^*E*^	*c*_13_^*E*^	*c*_33_^*E*^	*c*_44_^*E*^	*c*_66_^*E*^	*e*_15_	*e*_31_	*e*_33_	*ε*_11_^*S*^	*ε*_33_^*S*^
139	77.8	74.3	115	25.6	30.6	12.7	−5.2	15.1	370	635
